# The role of the occupational physician in the early detection of occupational diseases

**DOI:** 10.47626/1679-4435-2026-1564

**Published:** 2026-05-14

**Authors:** Lucas Fernando Lanfredi, Nathan Pires de Oliveira, Saulo da Silva Diógenes

**Affiliations:** 1 Faculdade Cetrus Sanar, Medicina do Trabalho, Brasília, DF, Brazil; 2 Escola Superior de Ciências da Saúde, Medicina, Brasília, DF, Brazil; 3 Universidade Federal do Ceará, Saúde Pública, Fortaleza, CE, Brazil

**Keywords:** occupational medicine, occupational diseases, early diagnosis, surveillance of the workers health.

## Abstract

Early detection of occupational diseases is essential for promoting health in the workplace, contributing to the prevention of injuries, reducing absenteeism, and improving the quality of life of workers. Occupational physicians play a strategic role in this process by identifying early signs and symptoms of diseases, assessing occupational risk factors, and collaborating with management in the implementation of preventive and corrective measures. This scoping review aimed to investigate the role of occupational physicians in the early identification of occupational diseases, considering their legal responsibilities, clinical practice, and interaction with interdisciplinary teams. Scientific evidence was synthesized regarding the most effective medical practices in this field, as well as the challenges faced by professionals in their daily work routine. The findings are expected to contribute to strengthening preventive actions in occupational health, informing occupational health policies, and supporting professional training focused on workers’ health surveillance.

## INTRODUCTION

Occupational health is a crucial component of public health, aimed at promoting the physical, mental, and social well-being of individuals in their work environment. Occupational diseases represent a significant group of work-related conditions, and their early detection is fundamental for timely intervention, appropriate rehabilitation, and prevention of further harm [^1^].

Historically, occupational medicine emerged during the Industrial Revolution in response to the marked increase in diseases and accidents associated with poor working conditions and has since undergone significant transformations. In Brazil, important regulatory frameworks have been established, such as the Consolidation of Labor Laws (*Consolidação das Leis do Trabalho*, CLT), the Regulatory Standards (*Normas Regulamentadoras*, NR), and the National Policy on Workers’ Health [^2^]. More recently, Ordinance GM/MS No. 5,201, dated August 15, 2024, expanded mandatory reporting of the main work-related diseases to all health services, whereas it had previously been limited to sentinel surveillance networks. These measures have officially recognized the importance of monitoring occupational risks and establishing prevention and health surveillance strategies in the workplace.

Within this context, the occupational physician responsible for the Occupational Health Medical Control Program (Programa de Controle Médico de Saúde Ocupacional, PCMSO), working in collaboration with the interdisciplinary team of the Occupational Safety and Health Service (Serviço de Engenharia e Medicina do Trabalho, SESMT) and with workers, is legally accountable for both active and passive health surveillance in the workplace, as established by NR 01. Accordingly, these professional plays a central role in identifying early clinical signs of occupational diseases, given their knowledge of the natural history of these conditions. Their responsibilities include conducting pre-employment, periodic, fit-for-work, and dismissal medical examinations, as well as assessing the causal relationship between occupational exposures and clinical conditions [^3^].

Evidence from scientific literature shows that early diagnosis of occupational diseases is directly associated with better clinical prognosis and lower functional and social impact [^4^]. However, many work-related diseases tend to develop insidiously and with subclinical symptoms, requiring a proactive and vigilant approach from occupational physicians during clinical assessments and periodic follow-up [^5^].

The main routes of exposure to harmful agents are the inhalation and dermal routes. The inhalation route is the main pathway affecting workers’ health, with approximately one in six cases of asthma and chronic obstructive pulmonary disease (COPD) being work-related [^6^]. Furthermore, occupational asthma is the most prevalent work-related lung disease in industrialized countries, accounting for about 15% of new asthma cases in adults. Its annual incidence ranges from 12 to 170 cases per million workers, with a prevalence of 5% to 15% across different sectors. It is characterized by variable airflow limitation or airway hyperresponsiveness to specific workplace exposures and is mainly caused by sensitizers (eg, animals, bioaerosols, medications, enzymes, latex, plants, seafood, acid anhydrides, metals, wood dust, persulfate, colophony, and isocyanates) and irritants (eg, chlorine, dust, high-level smoke, chemical vapors, pollution, and combustion products) [^7^].

Fibrogenic pneumoconioses, such as silicosis and asbestosis, also warrant attention. These are interstitial lung diseases caused by long-term inhalation of mineral particles, such as silica and asbestos, in occupational environments [^6^]. Unlike pneumoconioses, occupational asthma may be reversible if diagnosed early and if exposure to the causal agent is promptly eliminated [^7^].

The dermal route represents the second most relevant route of infection, with contact dermatitis being one of the most common occupational diseases. Risk factors such as working in wet environments and individual predisposition, as in the case of atopic dermatitis, are directly associated with its occurrence [^8^].

Occupational dermatitis accounts for approximately 90% of work-related skin disorders and is subdivided into two main forms: irritant contact dermatitis - about 80% of cases - and allergic contact dermatitis. In Brazil, up to 10% of workers are estimated to present some type of occupational dermatitis, although underreporting is common [^9^,^10^]. Worldwide, irritant contact dermatitis accounts for up to 90% of occupational dermatoses, with an incidence ranging from 0.5 to 1.9 cases per 1,000 workers per year, with the hands being the main affected area [^11^]. The main risk factors include contact with irritating or allergenic chemical agents and repeated exposure to moisture and friction [^12^,^13^].

Occupational dermatitis reduces quality of life and may lead to work absence [^10^]. In 2024, according to data from the National Institute of Social Security (Instituto Nacional do Seguro Social, INSS), 53 sick-leave benefits were granted for allergic contact dermatitis (L23), 18 for irritant contact dermatitis (L24), and 13 for unspecified contact dermatitis (L25), totaling 84 formal leaves among Brazilian workers. These data reflect the impact of dermatitis on workers’ quality of life by demonstrating the need for temporary work leave and its social and economic repercussions [^14^,^15^].

Irritant contact dermatitis results from the direct action of chemical or physical agents that damage the skin barrier, triggering an inflammatory response and producing symptoms such as erythema, burning, pruritus, vesicles, and, in long-term cases, lichenification. Allergic contact dermatitis, in turn, is a type IV immunological reaction mediated by T lymphocytes, occurring after prior sensitization of the individual to the allergen, leading to skin inflammation involving specific cytokines and resulting in eczematous lesions with poorly defined borders, clinically distinguished from the irritant form by the latency in symptom onset [^16^-^19^].

Accurate diagnosis of occupational dermatitis is dependent on a detailed patient history, careful clinical examination, and patch testing, in order to differentiate irritant from allergic forms, in addition to the assessment of environmental and occupational conditions. Objective criteria, such as the seven Mathias criteria for establishing occupational causation, assist in confirming the diagnosis. Occupational dermatitis may cause chronic skin changes that significantly impair workers’ quality of life, including fissures, lichenification, prolonged work absence, and job loss [^8^,^16^,^19^-^21^].

Overall, occupational diseases involve multiple pathophysiological mechanisms, characterized by chronic inflammatory processes and inadequate adaptive responses to repetitive exposures in the workplace [^22^]. In the case of work-related musculoskeletal disorders (WRMSDs), continuous mechanical overload of muscles, tendons, and peripheral nerves promotes sustained release of inflammatory mediators, including pro-inflammatory cytokines such as interleukin-1 and tumor necrosis factor-alpha, as well as prostaglandins and free radicals [^23^]. Repeated exposure and delayed intervention may lead to tissue fibrosis, long-term functional impairment, persistent pain, and reduced mobility, significantly affecting workers’ quality of life and work capacity [^24^]. Adequate management includes not only clinical treatment and physical rehabilitation but also ergonomic interventions, readaptation to work, and preventive policies in the workplace, in accordance with occupational health guidelines [^25^].

WRMSDs are one of the leading causes of occupational morbidity in Brazil and the world. In Brazil, from 2007 to 2019, more than 93,000 cases were reported, with higher prevalence among female workers in the Southeast and South regions, coinciding with the country’s main economic and industrial centers [^26^]. The prevalence of WRMSDs in the Brazilian adult population is approximately 2.5%, with regional variations reflecting socioeconomic and occupational factors [^27^]. Globally, in 2021, approximately 1.7 billion cases were recorded, with greater increases in lowand middle-income countries, particularly affecting women and individuals aged 50 to 59 years [^28^,^29^]. These disorders are mainly caused by biomechanical, organizational, and psychosocial factors, resulting in high social and economic burden, as well as work-related disability [^30^].

Another condition affecting workers’ health is noise-induced hearing loss (NIHL), resulting from prolonged and repeated exposure to elevated levels of occupational noise. This type of hearing loss is sensorineural, usually bilateral and irreversible, initially affecting high frequencies, especially in the range of 3,000 to 6,000 Hz [^31^]. The prevalence of NIHL varies according to occupation, country, and sex, being higher among men. This is because men are generally more exposed to high noise levels at work than women, due to differences in occupational categories, economic sectors of employment, and lifetime work history. Individuals aged 30 to 59 years are more vulnerable. Additionally, aggregated data indicate prevalences of up to 58% among construction workers [^32^].

In Brazil, between 2012 and 2021, 7,413 cases were reported, with higher prevalence among men aged 50 to 59 years, especially in the Southeast region [^33^]. Occupational noise exposure is responsible for 16% of disabling hearing loss cases in adults worldwide, corresponding to a significant burden of years lived with disability [^34^,^35^]. Prevalence varies across countries and sectors, being higher in developing nations due to the lower implementation of preventive measures, reaching approximately 47% among steel industry workers in some studies [^35^]. Globally, the burden of NIHL has shown a decreasing trend since 1990, particularly in regions with lower socioeconomic indices, but remains high, especially among middle-aged and older men [^34^].

Work-related mental disorders also deserve attention, as they may result from prolonged exposure to psychosocial stressors in the workplace, leading to dysfunction of the hypothalamic-pituitary-adrenal axis, with sustained elevation of cortisol and catecholamine levels, in addition to the activation of neuroinflammatory processes [^36^]. These alterations result in imbalances in the dopaminergic and serotonergic systems, contributing to the development of depressive disorders, anxiety, and chronic fatigue [^37^]. Furthermore, the List of Work-Related Diseases already documents several mental disorders associated with exogenous intoxications, especially those involving heavy metals and organic solvents.

Between 2018 and 2023, Brazil recorded 13,464 notifications of work-related mental disorders, showing a marked increase of 165% during this period. The highest number of cases was observed in 2023, with the Southeast region accounting for most cases (44.7%), reflecting regional socioeconomic disparities. Women were disproportionately affected, representing 68.06% of notifications, with the 35-44 age group being the most affected. Workers with higher educational levels comprised a significant proportion of cases, possibly due to greater professional demands and responsibilities. The overall prevalence of common mental disorders among Brazilian workers is estimated at around 30%, with some occupations, such as social educators, bank employees, teachers, and garbage collectors, showing even higher rates, reaching up to 58% [^38^,^39^].Clique ou toque aqui para inserir o texto.

Globally, approximately 970 million people were affected by mental disorders in 2019, an increase from the 654 million recorded in 1990. Among adults of working age, it is estimated that 15% had some type of mental disorder in 2019. Work-related mental disorders contribute significantly to productivity loss, with millions of workdays lost annually worldwide. Anxiety and depressive disorders are among the most common globally. The number of new cases of mental disorders worldwide reached approximately 444 million in 2021, highlighting the increasing burden of these conditions [^40^,^41^].

Therefore, the growing complexity of work environments and the diversification of exposure to occupational risks imply the need for occupational physicians to be knowledgeable about the main work-related diseases and to act preventively. Thus, the objective of this study was to identify articles highlighting tools and strategies for workers’ health surveillance that may support the action of occupational physicians in this process.

## METHODS

This study is a scoping review of the literature on the role of occupational physicians in the early detection of occupational diseases.

The research question was developed using the PICO framework, where: P (population) - occupational physicians; I (intervention) - involvement in the early detection of occupational diseases; C (comparison) - conventional approaches or absence of intervention; O (outcome) - effectiveness in early identification and prevention of adverse outcomes. Based on this framework, the following guiding question was formulated: “How should the occupational physician act in the early detection of occupational diseases, and what are the most effective practices for this purpose?”

The PubMed, Scopus, Web of Science, LILACS, and Biblioteca Virtual da Saúde databases were searched between May and August 2025 using descriptors in Portuguese and English related to the study topic, such as: “*medicina do trabalho*,” “*doenças ocupacionais*,” “*detecção precoce*,” “*prevenção*,” “*saúde do trabalhador*,” and their corresponding terms (“occupational medicine,” “occupational diseases,” “early diagnosis,” “prevention,” and “occupational health”). These descriptors were combined using Boolean operators (“AND” and “OR”) in pairs and trios to expand the search strategy. Filters for “occupational diseases” and “early detection” were applied, and only studies published from 2000 onward were included.

The six steps involved in conducting a scoping review were followed: (1) identifying the research question; (2) identifying relevant studies; (3) selecting studies to be included in the review; (4) data charting; (5) collating, summarizing, and reporting the results; and (6) consulting stakeholders - in this case, the third author of the study, an expert in the field who actively contributed to the development of the manuscript [^42^]. After that, the selected studies were exported, organized, and stored in Word spreadsheets to allow for duplicate identification and for the processes of study selection, inclusion, and exclusion.

**Figure 1:**
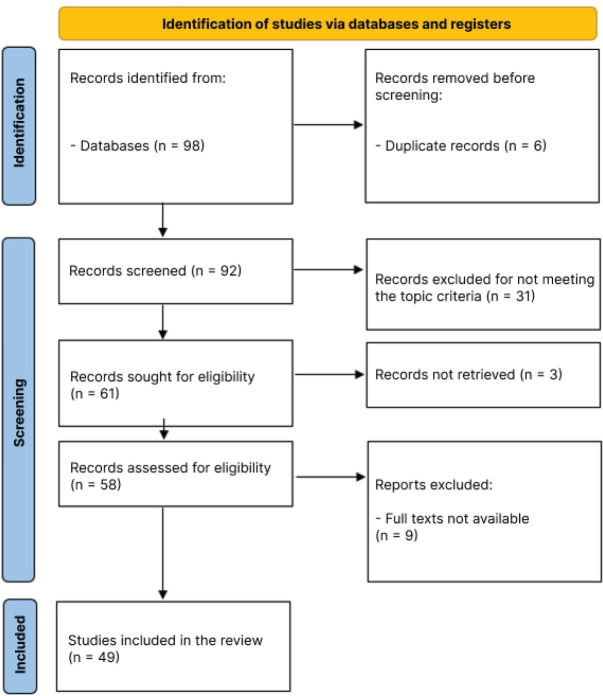
Flow diagram of study selection, Brasília (DF), Brazil, 2025. Source: Adapted from Page et al. [^6^]

Data extracted and organized in a Word document included information on author, year of publication, study design, population, interventions, outcomes, and main findings. Subsequently, a critical evaluation of the included studies was performed to identify effective practices, challenges, and existing gaps in the role of occupational physicians. The results were then analyzed and synthesized in Table 1, with the aim of contributing to the advancement of knowledge and the improvement of occupational health practices. The study selection process is reported in Figure 1, in accordance with the Preferred Reporting Items for Systematic Reviews and Meta-Analyses [^42^], which are also applicable to scoping reviews.

**Table 1:** Summary of included studies on the role of the occupational physician in the early detection of occupational diseases

Article and study location	Objective	Study type/design	Summary of results
Howlett et al. - UK [^6^]	To guide early recognition and management of pneumoconioses in the UK.	Qualitative study	Differentiated occupational asthma from work-aggravated asthma and highlighted the use of specific diagnostic tests for early detection, such as immunological tests (serum-specific IgE or skin-prick tests), detailed serial peak flow measurement, and specific inhalation challenge testing at specialist centers.
Chabra & Gupta - USA [^7^]	To outline the diagnosis, management, and treatment of allergic, environmental, and occupational asthma.	Qualitative study	Emphasized strategies for preventing exacerbation and the importance of the interprofessional team in improving patient care. Tools such as the asthma APGAR (activities, persistent, triggers, asthma medications, response to therapy) allow monitoring of disease severity and early identification of work-related exacerbations.
Dalbøge et al. - Denmark [^43^]	To identify and assess the relationship between 10 potential occupational sensitizing exposure groups and the development of asthma to support diagnosis, prevention, and regulatory policies.	Qualitative study	Questionnaires, although commonly used in observational studies, are low-confidence methods with potential risk of bias and misclassification. In contrast, the review was positioned as an essential tool to support workplace risk assessments. In practice, occupational physicians can use these findings to identify hazards, assess risks - particularly frequent exposures such as pesticides or epoxy - and promote preventive actions.
Hasan et al. - Bangladesh [^36^]	To improve early detection of occupational stress using machine learning methods and large language models, providing a real-time accessible and scalable monitoring tool.	Quantitative study	Proposed stress detection without biomarkers, based solely on questionnaire responses, with potential for periodic and real-time application.
Strudwick et al. - Australia [^37^]	To evaluate the efficacy of workplace mental health screening programs on employee mental health, productivity, adherence, quality of life, help-seeking behavior, and adverse effects.	Mixed-methods study	Found that screening alone has limited effectiveness and highlighted the need for further studies assessing the combined impact of interventions.
Balachandar et al. - India [^44^]	To develop occupational mental health screening tools focused on burnout and psychosocial factors among the Indian workforce.	Quantitative study	Developed two scales: one for burnout (emotional exhaustion, depersonalization, and personal accomplishment) and another for work environment (workload, reward, and cooperation).
Van Wijk et al., 2022 - South Africa [^45^]	To propose a concise mental health screening tool, based on existing data, for routine occupational health surveillance in South Africa.	Quantitative study	Demonstrated potential for concise screening (<50 items), with robust predictive analysis and applicability for both clinical and organizational interventions.
Linton - Sweden [^22^]	To use psychological factors as risk predictors and evaluate the effectiveness of cognitive-behavioral interventions.	Quantitative study	Highlighted the need for early interventions to prevent long-term work-related musculoskeletal pain. In mediumand high-risk patients, cognitive-behavioral intervention significantly reduced work disability compared to usual care, with no significant difference in the low-risk group.
Soares et al. - Brazil [^23^]	To discuss the effectiveness of workplace physical exercise programs as a form of primary prevention.	Qualitative study	Highlighted the high prevalence of WRMSDs and their negative impact on quality of life and associated costs. Workplace physical exercise programs, when properly planned and tailored to workers’ profiles, were shown to be effective in the primary prevention of WRMSDs. Consideration of intensity, frequency, and individual adaptation was necessary to achieve optimal outcomes.
Ekhammar et al. - Sweden [^24^]	To investigate health care professionals’ experiences with the PREVSAM tool for early rehabilitation of patients with musculoskeletal disorders at risk of work absence, particularly their perceptions of its clinical benefit and feasibility.	Qualitative study	The PREVSAM tool was perceived as beneficial for patients, professionals, and institutions, promoting person-centered care and team-based rehabilitation. Implementation barriers were identified, requiring support from managers and policy makers.
Sweileh - Palestine [^25^]	To map global scientific production on WRMSDs, focusing on risk factors and preventive approaches, through the analysis of publications indexed in the Scopus database between 1993 and 2022.	Quantitative study	Recommended integrative and multidisciplinary preventive actions, promoting collaboration between occupational physicians and other team sectors. These included investigating the effectiveness of preventive interventions; encouraging interdisciplinary research involving psychology, ergonomics, rheumatology, and occupational health; exploring the role of technologies, such as wearable devices, in reducing WRMSDs (eg, PPE with real-time movement alerts for construction workers); and efforts at the managers’ level to translate scientific evidence into practices and policies that ensure occupational safety and health.
Mirza et al. - USA [^31^]	This position statement of the American College of Occupational and Environmental Medicine aimed to describe best practices for the diagnosis and prevention of ONIHL, highlighting the role of the occupational physician in its prevention, early diagnosis, and management.	Qualitative study	Identified audiometric testing as essential for early detection. Emphasized the essential role of the occupational physician in identifying occupational, nonoccupational, chemical, and individual factors that contribute to hearing loss, as well as the importance of detailed history, adequate PPE use, and surveillance for progressive hearing loss.
Chen et al. - China [^32^]	To present a synthesis of global prevalence data by occupation and country and to discuss physiological and environmental factors associated with ONIHL.	Mixed-methods study	Prevention, particularly through noise control programs, was identified as the most effective measure, pending validation of treatments such as antioxidants.
Samelli et al. - Brazil [^46^]	To evaluate the effectiveness of interventions aimed at preventing occupational hearing loss, such as hearing conservation programs and audiological monitoring.	Mixed-methods study	Demonstrated positive outcomes with training on PPE use and hearing protection, as well as increased PPE use and reduced noise levels through hearing conservation programs.
Jogie - Trinidad and Tobago [^16^]	To address chemical and physical risks affecting paint workers and to integrate occupational medicine, clinical medicine, and primary care approaches to guide preventive and therapeutic strategies.	Qualitative study	Identified effective preventive strategies, including adequate ventilation, adequate PPE use, routine clinical screening, monitoring, and continuous worker education, emphasizing the importance of an interdisciplinary approach to protecting occupational health.
Awodele et al.- Nigeria [^19^]	To assess the use of available control measures in paint factories using a standardized WHO questionnaire and urinary heavy metal analysis in exposed workers.	Quantitative study	Among 400 workers, 72.5% were aware of occupational risks, only 30% had received formal training, and 40% did not use PPE. Approximately 90% reported symptoms related to occupational exposure. These findings highlight the need for occupational physicians to require immediate implementation of formal training for the remaining workers and to actively promote and monitor PPE use during periodic examinations to address nonadherence.
Vearrier & Greenberg - USA [^47^]	To discuss the applicability of medical monitoring following occupational or environmental exposure to toxic agents.	Qualitative study	Advocated an approach based on scientific criteria, such as the Bradford Hill criteria, to assess causality and risk magnitude.
Schütte et al. - Netherlands [^8^]	To identify occupational and personal risk factors for contact dermatitis and evaluate their association with the disease, in order to assess the importance of prevention.	Quantitative study	Concluded that multiple occupational and individual factors are associated with contact dermatitis, reinforcing the importance of combined risk assessment for prevention.

IgE = immunoglobulin E; NIHL = noise-induced hearing loss; PPE = personal protective equipment; PREVSAM = Prevention of Sickness Absence Through Early Identification and Rehabilitation of At-Risk Patients with Musculoskeletal Disorders; WHO = World Health Organization; WRMSDs = work-related musculoskeletal disorders.

Original studies, systematic reviews, and other scientific publications that clearly and objectively addressed the role of occupational physicians in the early identification of occupational diseases were included, with no restrictions regarding language or publication period. Manuscripts focused solely on administrative or organizational aspects, without a clinical approach, were excluded, as well as studies based exclusively on secondary data without adequate methodological description, such as opinion pieces or articles that had not undergone peer review.

The protocol of this scoping review was registered on the Open Science Framework platform (https://doi.org/doi.org/10.17605/OSF.IO/C9HFE), in order to ensure methodological transparency and reproducibility of results.

## RESULTS

After screening and study selection, 18 articles were included, and their synthesis is presented in Table 1, categorized according to disease groups.

## DISCUSSION

The findings of this scoping review highlight the strategic role of occupational physicians in the early detection of occupational diseases, emphasizing their contribution to workers’ health surveillance, organizational management, and clinical intervention. The included studies, organized by disease categories, demonstrate that early, interdisciplinary, and evidence-based medical practice plays a key role in mitigating adverse health outcomes among workers.

Regarding occupational lung diseases, the review by Howlett et al. [^6^] emphasizes the high prevalence of conditions such as occupational asthma and COPD, underscoring the importance of a detailed clinical history for early diagnosis. The study reported that approximately 1/3 of patients with occupational asthma become unemployed within 3-5 years, highlighting the need for early medical intervention by occupational physicians. The investigation of occupational asthma requires specific testing performed by a specialist, including immunological tests (serum-specific IgE or skin-prick tests) to confirm sensitization to causal agents. Detailed serial peak flow measurement, with measurements recorded at least four times a day over 3 consecutive weeks, was identified as the most accessible diagnostic tool.

Complementing these findings, the study by Dalbøge et al. [^43^] identified moderate evidence for emerging sensitizing agents, including crustaceans, enzymes, pesticides, and cleaning products, based on an analysis of 55 studies covering 10 occupational exposure groups. These results highlight the importance of interprofessional collaboration and continuous updating on emerging sensitizers, suggesting that the training of occupational physicians should include not only advanced clinical competencies but also communication and risk management skills.

Regarding mental health in the workplace, studies such as that by Hasan et al. [^36^] demonstrate the potential of machine learning methods for early screening of occupational stress, although adequate organizational infrastructure is required for their practical implementation. In this study, an ensemble-based model integrating three algorithms achieved an accuracy of 90.32% in detecting occupational stress, with the main associated factors being excessive workload and ambiguity (27%), poor communication (17%), and a positive work environment (16%). In this context, occupational physicians may also rely on validated instruments, such as the Work Stress Questionnaire (WSQ), which assesses multiple domains, including work demands, job control, and organizational support.

Conversely, Strudwick et al. [^37^] showed that screening alone has limited effectiveness. In this study, screening followed only by advice or referral was ineffective in improving mental health symptoms (d = -0.07), while screening followed by facilitated access to treatment showed small improvements (d = -0.22). These findings highlight the need for occupational physicians to work in coordination with psychologists, managers, and institutional policies to ensure that early detection is accompanied by effective therapeutic interventions.

In the study by Balachandar et al. [^44^], the developed tools demonstrated adequate internal consistency (α = 0.82 and 0.73) and a statistically significant correlation with a general mental health scale. Based on a sample of 2,303 participants, Van Wijk et al. [^45^] identified 14 markers organized into five domains: neurocognitive health, common mental disorders, history of adaptation in occupational-specific contexts, work-family interface, and stress overload.

To minimize response bias and increase applicability across different professions, a Brazilian study demonstrated that the Oldenburg Burnout Inventory (OLBI) showed good fit to the national sample. The OLBI is a 16-item instrument comprising two core dimensions of burnout - exhaustion and disengagement from work - each assessed using a Likert-type scale. The final score reflects the level of emotional exhaustion and disengagement from work activities, including both positively and negatively worded items. The instrument showed a sensitivity greater than 80% for the identification of occupational burnout, allowing the occupational physician a more objective assessment. It can be used both for early screening and for longitudinal monitoring of workers exposed to psychosocial risk factors. Its application allows occupational physicians to identify burnout at early stages, support targeted interventions, and monitor the effectiveness of mental health prevention and promotion programs in the workplace [^48^].

Additionally, as highlighted in the studies by Balachandar et al. [^44^] and Van Wijk et al. [^45^], culturally adapted tools reinforce that there are no universal solutions and that local context must be considered when implementing screening protocols.

Regarding WRMSDs, the included studies indicate that early intervention by occupational physicians, including behavioral interventions, exercise programs, and rehabilitation, has a direct impact on preventing work absence and improving workers’ quality of life. This approach requires tailoring workplace exercise programs to individual needs, which depends on careful and continuous medical evaluation. The effectiveness of these strategies in preventing absenteeism is supported by early identification and rehabilitation of at-risk patients, using models such as Prevention of Sickness Absence Through Early Identification and Rehabilitation of At-Risk Patients with Musculoskeletal Disorders (PREVSAM), as well as cognitive interventions and screening tools such as the Örebro Musculoskeletal Pain Screening Questionnaire-Short Form (ÖMPSQ-SF), which can modify the trajectory of chronic pain and disability. For functional monitoring, occupational physicians may use validated instruments such as the Short Musculoskeletal Function Assessment (SMFA), which demonstrates excellent internal consistency and stability, with values greater than 0.90 [^22^-^24^]. The PREVSAM model is based on a biopsychosocial perspective with a person-centered approach that includes individual assessments and structured rehabilitation delivered by an interdisciplinary team. In the study by Sweileh [^25^], which analyzed 1,132 articles on WRMDs, low back, neck, and shoulder pain were identified as the most commonly affected body regions. Occupational physicians may also implement tools such as the Musculoskeletal Health Questionnaire (MSK-HQ), which allows standardized monitoring of symptoms and quality of life, as well as ergonomic assessment instruments such as Rapid Upper Limb Assessment (RULA), Occupational Repetitive Actions (OCRA), and Hand Activity Level (HAL), for the objective identification of risk factors.

NIHL is one of the most prevalent occupational diseases worldwide. Studies highlight the lack of robust evidence on long-term interventions, underscoring the need for greater national and international scientific production on this topic [^32^,^46^]. The review by Mirza et al. [^31^] emphasizes the role of occupational physicians not only in early detection, through periodic audiometry, but also in continuous surveillance of environmental conditions and adherence to hearing protection measures. In this context, occupational physicians play a key role in implementing audiometric protocols, including pure-tone audiometry - the gold standard for NIHL assessment - and complementary tests such as otoacoustic emissions or speech-in-noise testing, which may detect early subclinical damage.

Accordingly, occupational physicians should establish hearing conservation programs, including baseline and annual audiometric evaluations for workers exposed to noise levels ≥ 85 dB(A) over an 8-hour workday. The identification of a significant change in the hearing threshold, defined as an average change of ≥ 10 dB at 2, 3, and 4 kHz, requires immediate action, including reassessment of hearing protection and referral to a specialist [^49^].

The onset of NIHL is often silent and insidious, with early symptoms including difficulty understanding speech in noisy environments and muffled hearing. Although disease progression can be prevented by eliminating or reducing exposure to safe noise levels, the damage is irreversible once established, reinforcing the importance of primary prevention. Preventive measures should prioritize elimination of the hazard and, when not feasible, the implementation of collective protective measures, such as placing machinery inside enclosures, as well as administrative and organizational strategies, such as reducing exposure time through job rotation. If residual noise persists, personal protective equipment may be used, in accordance with NR 01. Environmental noise control and periodic audiometric surveillance are essential for prevention [^46^].

In the field of occupational dermatitis, studies emphasize that early detection should rely on rigorous clinical screening combined with continuous monitoring of workers’ health, with particular attention to high-risk groups such as workers in the paint industry, health care, cleaning services, and other occupations involving frequent exposure to irritants and sensitizers. In the study by Larese Filon et al. [^20^], the incidence of occupational contact dermatitis ranged from 0.6 to 6.7 per 10,000 person-years in registry-based studies, whereas cohort studies reported higher incidences, from 15.9 to 780 per 10,000 person-years, particularly among apprentice nurses and dentists. Similarly, Schütte et al. [^8^] found moderate evidence for the association between wet work and irritant contact dermatitis (odds ratio 1.56; 95%CI 1.21-2.01), and strong evidence for the association between pre-existing atopic dermatitis and irritant contact dermatitis (odds ratio 2.44; 95%CI 1.89-3.15).

In the study by Awodele et al. [^19^], among 400 paint factory workers, 72.5% were aware of occupational risks, yet only 30% had received formal safety training. Additionally, 40% did not use personal protective equipment, and approximately 90% reported symptoms related to occupational exposure, highlighting a significant gap in prevention and risk management. Complementing this perspective, Jogie [^16^] underscores the importance of integrating occupational medicine, primary care, and specialist services to prevent systemic health effects associated with recurrent exposure, emphasizing the need for early intervention and an interdisciplinary approach.

In parallel, Vearrier & Greenberg [^47^] examined the appropriateness of implementing medical monitoring programs following potentially hazardous occupational, environmental, or pharmaceutical exposures in situations where a causal relationship has been established clinically or supported by the Bradford Hill criteria - namely strength of association, consistency, specificity, temporality, biological gradient, plausibility, coherence, experiment, and analogy. The authors emphasize that such programs should only be implemented when benefits outweigh risks and costs, requiring criteria such as documented exposure of sufficient severity to increase the risk of adverse effects, availability of sensitive and specific screening tests, feasibility of early disease detection, and, most importantly, evidence that early detection could reduce morbidity or mortality.

## CONCLUSIONS

The findings of this review reinforce the role of occupational physicians as professionals in a strategic position for the early identification of occupational diseases, playing a key role in promoting health and preventing adverse outcomes in the workplace. Their practice extends beyond individual clinical assessment, encompassing educational, epidemiological, legal, and interdisciplinary actions.

The results suggest that the effectiveness of early detection is directly linked to technical training, interdisciplinary practice, and the integration of surveillance, care, and organizational management. However, institutional barriers, structural limitations, and underreporting remain significant challenges, particularly in lowand middle-income countries, and were not fully addressed by the studies included in this review.

Study limitations include the qualitative nature of the scoping review, which does not incorporate meta-analysis, and the potential underreporting of occupational diseases in the literature. Additionally, heterogeneity among health care systems across countries limits the generalizability of the proposed practices to different institutional contexts. Despite these limitations, this study presents innovative and effective approaches for the early detection of occupational diseases based on the reviewed literature, addressing the guiding research question, although not exhaustively, given the methodology and scope of the study. Future research focusing on specific groups of occupational diseases is recommended to allow a more in-depth analysis of early detection practices, which will be explored in subsequent studies by the authors.

Further longitudinal and intersectoral research is needed to assess the long-term impact of medical interventions, as well as the effectiveness of screening tools across different contexts. Strengthening the training of occupational physicians, together with the expansion of public policies focused on occupational health, is essential to consolidate a more robust, evidence-based preventive model aimed at preserving work capacity and promoting workers’ well-being.

## Data Availability

Upon publication, the data will be made available by the authors upon request.
